# Manipulation of THC Hair Concentrations by Commercially Available Products

**DOI:** 10.3390/metabo12100900

**Published:** 2022-09-24

**Authors:** Markus Bertges, Alexandra Ketikidou, Ralf Weiskirchen, Josef van Helden, Rudolf Boehnke, Cornelius Hess

**Affiliations:** 1 MVZ Dr. Stein + Kollegen Mönchengladbach, Labor Mönchengladbach, Medical Care Centre, D-41169 Mönchengladbach, Germany; 2 Institute of Molecular Pathobiochemistry, Experimental Gene Therapy and Clinical Chemistry (IFMPEGKC), RWTH University Hospital Aachen, D-52074 Aachen, Germany; 3 Immunlab-Gemeinschaftspraxis für Laboratoriumsmedizin Dr. med. Adamek und Dr. med. van Helden GbR, D-40476 Düsseldorf, Germany; 4 TOXILAB Ludwigsburg, Labor für Toxikologie und Drogenuntersuchungen GmbH, D-71636 Ludwigsburg, Germany

**Keywords:** drug screening, hair, manipulation, tetrahydrocannabinol, hair care products, bleaching, coloring, forensics

## Abstract

Many drug tests are carried out by means of hair analysis. The aim of the present study was to clarify if and to what extent it is possible to manipulate the results of hair analyses on tetrahydrocannabinol (THC) by using various commercially available everyday products and products advertised on the internet to be able to reduce the concentrations of drugs in hair. Fifty-four THC-positive hair samples were analyzed using liquid chromatography tandem mass spectrometry; they were analyzed untreated or treated with Vodka Gorbatschow^®^ (n = 19), Seborin^®^ hair tonic (n = 11), Zydot^®^ shampoo (n = 6), Desderman^®^ disinfectant (n = 11) and Head and Shoulders^®^ shampoo (n = 7). A mean reduction of 52% (Zydot^®^ shampoo) to 65% (Desderman^®^) was shown. Hair treatments could not be detected visually. Hair concentrations could also be decreased to non-detectability by using these everyday hair care products. Therefore, it is recommended to complement abstinence controls using hair samples by urine analysis and to not over-interpret quantitative results of THC concentrations in hair.

## 1. Introduction

In Germany, a forensic-based approach concerning driving under the influence of drugs involves drug testing procedures as part of a driving license re-granting process. Subjects have to prove abstinence by undergoing random (24 h notice) urine tests or, alternatively, by hair analyses covering six months or a whole year [1]. For tetrahydrocannabinol (THC), the recent analytical concentrations a laboratory needs to be able to detect in hair (i.e., 0.02 ng/mg) for this purpose are quite low [1]. The guidelines of the Society of Hair Testing (SoHT) recommend a cut-off to identify the use of 0.05 ng THC/mg hair [2]. German laboratories which use more sensitive methods are allowed to report positive results even for <0.02 ng THC/mg hair [1]. However, due to the issue of passive contamination and due to equality reasons, future guidelines are discussed to set a clear negative/positive cut-off at 0.02 ng THC/mg hair, and positive results < 0.02 ng THC/mg hair are reported as negative results.

The composition of hair is mainly protein (may range from 65 to 95% (keratin)), with 1–9% lipids, 15–35% water and less than 1% minerals [3]. The average hair growth is 0.60–1.42 cm/month and there are several established techniques used to analyze drugs [4]. The hair always contains a mixture of anagen (growth phase), katagen (transition phase) and telogen (failure phase) [3]. In this mixture, an entry of substances from the katagen and telogene hair can arise from time-shifted temporal ranges [4]. These things have been known for a long time and forensic toxicologists can deal with them very well. The incorporation rate of cannabinoids in hair via blood is mostly dependent on the time at which cannabinoids are available in the bloodstream. The melanin content of the hair is also of significant importance for drug incorporation, but far less for cannabinoids due to its acidity [5].

Hair analysis can be further complicated due to cosmetic hair treatments. Bleaching causes oxidative damage and degradation of melanin granules due to high concentrations of hydrogen peroxide [6]. Permanent dyeing of hair using alkali solutions and low concentrations of hydrogen peroxide or temporary dyeing with colored dyes is also frequently used to achieve a coloring effect [6,7]. The coloring and bleaching of hair have been shown to be able to reduce THC concentrations by 30% or 14%, respectively [8]. Another study showed a decrease in THC concentrations after bleaching by 34% (mean, range: −16.1% to −65.7%; n = 15) and a decrease in THC concentrations after perming by 48.2% (range: −24.2% to −74.8%; n = 10) [9].

Due to these reductions in concentrations, negative findings in bleached or colored hair are only allowed to show abstinence in the German driving license re-granting process if the hair analysis is complemented by additional urine analyses [1]. Bleaching or coloring is visible to the analyzing laboratory for the most part, however, there are everyday products and treatment procedures controversially discussed in internet forums which are not easily detectable by the eye but could also be able to reduce cannabinoid concentrations.

The aim of this study was to provide more information on the effect of these treatments on THC concentrations in hair. We have chosen the following over the counter products: Seborin^®^ hair tonic, vodka Gorbatschow^®^, Zydot^®^ shampoo and Head and Shoulders^®^ shampoo.

## 2. Materials and Methods

### 2.1. Chemicals

All solvents (dichloromethane, methanol, ammonium acetate, ammonia (25%) and acetic acid (100%)) were supplied by VWR (Langenfeld, Germany). THC (1 mg/mL) and THC-d3 (0.1 mg/mL) reference standards were bought from LGC Standards (Wesel, Germany).

### 2.2. Hair Samples

THC-positive hair samples were chosen anonymously from our routine database. Only positive hair samples in which no chemical hair treatment by bleaching, perming or coloring was mentioned or visually detectable were chosen. However, we cannot completely rule out the possibility that the hair was treated before our study. Hairs from 54 individuals were taken from the back of the head. Hair strands were divided into 2 separate locks: one part was kept as its own control, while the second strand was subjected to the assigned hair treatment: 19 hair samples with vodka Gorbatschow^®^ (Berlin, Germany), 11 hair samples with Seborin^®^ hair tonic (Schwarzkopf, Düsseldorf, Germany), 6 with Zydot^®^ shampoo (Nuremberg, Germany), 11 with Desderman^®^ disinfectant (Schülke & Mayr GmbH, Norderstedt, Germany) and 7 with Head and Shoulders^®^ shampoo (Procter Gamble, Cincinnati, OH, USA).

Additionally, thick strands of hair taken from 4 test persons known to regularly consume cannabis were cut into 5 parts and analyzed without pre-treatment and subsequently with all four treatment procedures. The hair samples were taken from the following donors: P1 (44 years old, male, full black/grey hair), P2 (27 years old, male, full black hair), P3 (25 years old, male, full dark blond hair) and P4 (25 years old, male, full red/brown hair), respectively.

### 2.3. Cosmetic Treatment 

Treatment was carried out in vitro using the following commercially available products: (i) vodka Gorbatschow^®^ (Lot-L1336, 37.5% ethanol); (ii) Seborin^®^ hair tonic (Lot-7115Z12038), (iii) Zydot^®^ shampoo (Detox Shampoo & Conditioner Kit), (iv) Desderman^®^ disinfectant (Ch.B-1512068, 78.2 g Ethanol/96%, 0.1 g Biphenyl-2-ol) and (v) Head and Shoulders^®^ shampoo (Lot-91624224). All products were applied on the hair samples using the following procedure: The full length of the hair strand was washed three times with the respective agent (each for 30 min). Each washing cycle was followed by 10 min of washing with distilled water. The samples were placed on an overhead rotator during the washing procedure. After the three washes, the hair samples were dried under a gentle stream of nitrogen. Afterwards, the proximal 3–6 cm (depending on the hair length) of the hair strand was taken to routine sample preparation. 

### 2.4. Sample Preparation

The THC-positive and pre-treated hair samples were washed (30 s in an Elma T450 ultrasonic bath (Elam Ultrasonic, Singen, Germany) with dichloromethane), dried (for 8 min at 70 °C in a dry oven) and cut into very small pieces using scissors (approx. 1–2 mm snippets). A maximum of 50 mg and a minimum of 30 mg of hair from each sample were weighed into a silanized headspace vial. The silanization should prevent the adhesion of THC to the vial wall. Subsequently, 50 µL internal standard solution (including THC-d3 at a concentration of 500 ng/mL) and 1 mL methanol were added. The headspace vials were then placed in an ultrasonic bath with cooling (<17 °C) for four hours. After extraction, each sample was filtered through a syringe attachment with a pore diameter of 0.45 µM into silanized test tubes (15 mL). Finally, the filtrates were evaporated to a dry state under a gentle stream of nitrogen and diluted with 300 µL buffer solution. The buffer solution consisted of a mixture of 400 mL methanol and 100 mL solution 1 (374 µL ammonia 25% in 500 mL water) set to pH 8 using acetic acid. The samples were mixed for 10 s and transferred to a vial. Finally, the samples were centrifuged, and the supernatant was used for LC-MS/MS analysis.

### 2.5. LC-MS/MS Analysis

#### 2.5.1. Analytical Method

A procedure was used for the detection of illicit drugs and their metabolites in hair, applying a liquid chromatography tandem mass spectrometry (LC-MS/MS) method. The equipment consisted of a Shimadzu liquid chromatograph (LC-20-AD/-SIL-HTC) and a Thermo Fisher TSQ Quantum Ultra Mass Spectrometer (Thermo Fisher Scientific, Meerbusch, Germany). The analytical column used was a ReproSil-Pur C18 column (125 × 2 mm; 5 µm; Techlab, Braunschweig, Germany) with a Luna C18 (20 × 2 mm; 10 µm) guard column (Phenomenex, Aschaffenburg, Germany). Mobile phase A was water (with 10 mmol/L ammonium acetate) and mobile phase B was methanol (with 10 mmol/L ammonium acetate). The gradient and flow rate was as follows: 0–2 min: 0% mobile phase B and 0.25 mL/min; 2–3 min: 0% mobile phase B and 1.5 mL/min; 3–13 min: 95% mobile phase B and 0.2 mL/min; 13–14 min: 0% gradient B and 1.5 mL/min (re-equilibration). Analytes were detected in the multiple reaction monitoring (MRM) mode with positive electrospray ionization. The following ion transitions were used: THC: m/z 315 ›193 (target; collision energy: 21 eV); m/z 315 › 259 (qualifier; collision energy: 17 eV); m/z 315 › 123 (qualifier 2; collision energy: 32 eV); THC-d3: m/z 318 › 196 (collision energy: 22 eV).

#### 2.5.2. Data Validation

The method was validated according to the Guideline of the German Association of Toxicology and Forensic Chemistry [10]. Validation data were the following for THC: limit of detection: 0.005 ng/mg; limit of quantification: 0.016 ng/mg. The linear range was 0.02 ng/mg to 0.4 ng/mg using six calibrators (i.e., 0.02 ng/mg, 0.08 ng/mg, 0.16 ng/mg, 0.24 ng/mg, 0.32 ng/mg and 0.4 ng/mg). Two quality controls at 0.024 ng/mg and 0.3 ng/mg were both used at the beginning and the end of each series and again after 20 consecutive samples within a series. Precision data were the following: bias 11.5% at 0.024 ng/mg and 6.7% at 0.3 ng/mg; intra-day precision 9.7% at 0.024 ng/mg and 4.0% at 0.3 ng/mg; and inter-day precision 11.6% at 0.024 ng/mg and 6.8% at 0.3 ng/mg. Bias and precision data were calculated from two measurements each for eight consecutive days. Matrix effects were shown to be 104% at 0.024 ng/mg and 105% at 0.3 ng/mg. Matrix effects caused by ingredients of the products extracted to the final solution were checked via comparison of the peak areas of the internal standard THC-d3 in the respective chromatograms of untreated and treated samples. There were no relevant differences in the peak areas of THC-d3.

### 2.6. Statistical Analysis

For the comparison of treated and untreated hair samples, a test for normal distribution and a Mann−Whitney U test (using Ho, H1) was performed using the software SPSS (IBM Corp., SPSS Statistics for Windows, Armonk, NY, USA). Therefore, all results < LoQ were taken as 0 ng/mg. Statistical significance was assumed when *p* was < 0.05.

### 2.7. Ethics

The samples of this study were taken due to a clinical indication and examined within the framework of quality management for the introduction of a new method or to increase the quality of diagnostics. The use of leftover samples together with data from routine clinical practice submitted with the lab order is covered in North Rhine-Westphalia (NRW) by the Data Protection Act NRW §6 “*Datenverarbeitung für wissenschaftliche Zwecke Abs. (2) und Abs. (3)*.” During data handling, all personal information was anonymized. Therefore, no separate ethics vote was required for this study.

## 3. Results

### Chemical Toxicological Hair Analysis

**Figure 1:**
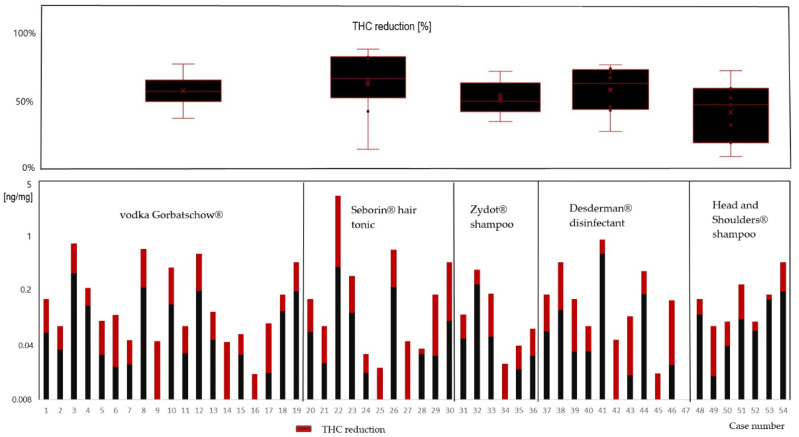
Comparison of hair extraction results without (black + red) and with (black) pre-treatment with the depicted over-the-counter products (decreased concentration in red).

Figure 1 shows the measured THC concentrations of the 54 hair samples without and with treatment with the described brands. Noteworthily, all the products that we selected had an impact on the concentration in the hair. 

A mean reduction of 58% by application of the vodka, 63% by Seborin^®^ hair tonic, 52% by Zydot^®^ shampoo, 65% by Desderman^®^ disinfectant and 52% by Head and Shoulders^®^ shampoo was shown. The reduction was significant (Mann−Whitney U test, significance was assumed when *p* < 0.05) for the use of vodka (*p* = 0.012), hair tonic (*p* = 0.022) and disinfectant (*p* = 0.047). However, the reduction of concentrations after treatment with Zydot (*p* = 0.115) and Head and Shoulders (*p* = 0.100) was not significant. The individual measured concentrations in each hair sample are also shown in Table 1.

**Figure 2:**
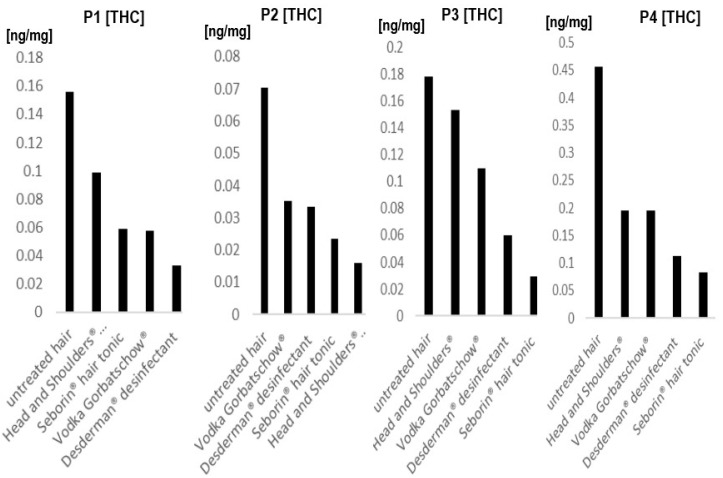
Tetrahydrocannabinol (THC) concentrations before and after treatment with different products in 4 different individuals. Each Figure shows one individual whose hair was treated in vitro with 4 different products and compared to an untreated sample. P1-P4, donor 1-donor 4. For details about hair samples, refer to 2.2.

Figure 2 shows the results of the 4 patients that regularly consumed cannabis whose hair underwent all 4 of the tested treatments. Results show that it depends on the individual hair sample which product leads to the highest decrease in concentrations, with Seborin^®^ hair tonic and Desderman^®^ disinfectant being the two products which seemed to lead to the highest decreases in THC concentrations. However, in hair sample 2, Head and Shoulders^®^ shampoo demonstrated the best results.

## 4. Discussion

In this study, we showed results on hair treatments with different over the counter products on THC hair concentrations. Bleaching, perming and coloring have shown to be able to reduce THC concentrations in the past [8,9]. However, these hair treatments are mostly visible for the person collecting the hair sample or during the sample preparation process. The treatments used here were not visible by eye and, therefore, they could easily be used by an accused to manipulate their hair sample results. Furthermore, the decrease in concentrations was even higher compared to those described in bleached or colored hair [8,9].

A reduction in THC concentrations by more than 50% on average is possible with every product and strongly depends on the individual hair condition. In our test series, three successive extractions were simulated. If used daily, this could lead to far higher decreases in THC concentrations. In 14 of the 54 cases shown here, the treatment led to concentrations < 0.02 ng/mg and thus would have been reported negative according to planned German guidelines or would have been decreased to concentrations lower than the limits of detection of the analytical methods.

The decrease in the concentrations was far higher than the measurement uncertainty of the used analytical method. It had to be taken into account that due to different percentages of hairs in anagen, katagen and telogen phases and due to other reasons, two close hair strands taken from the same individual at the same time do not have to have the same concentrations. Moreover, the hair samples from all the participants showed the same trend and even a significant reduction was shown.

Nevertheless, the percentage of decrease in cannabinoids varied between samples. This could be explained by a difference in the different hair specimens (thickness, porosity). The percentage of loss in concentration, however, was not dependent on the initial concentration of THC.

In the case of doubts surrounding the active consumption of cannabis products, it is recommended to analyze THC carboxylic acid in hair. Detection in very low concentrations is supposed to prove active consumption and metabolism. THC-COOH seems to be more stable than THC during the bleaching or coloring process [8]. However, the procedures described here also extract cannabinoids from hair and not only chemically change them. Therefore, we expect THC carboxylic acid concentrations are decreased like THC concentrations are, however, to a smaller extent due to its reduced lipophilicity. Results on changes in the THC-COOH concentration in hair will be presented elsewhere. However, since German laboratories usually analyze THC only and an analysis on THC-COOH is only added if a positive THC hair result is suspected, negative THC results (in the case of manipulation by the use of these products) would not be checked by an additional THC-COOH analysis.

In addition, we also expect smaller decreases of less lipophilic drugs in hair (amphetamines, cocaine) compared to THC when using these treatments. Future studies should show how concentrations of other drugs of abuse are changed during these treatments.

Overall, these results indicate that everyday products like shampoos or hair tonics (Seborin^®^ hair tonic) or Head and Shoulders shampoo^®^), which do not even have to be used to intentionally reduce cannabinoid concentrations, can lead to lower concentrations and the non-detectability of drugs. Treatments intentionally used like Zydot^®^ shampoo or ethanolic solutions/beverages like vodka or disinfectant did not lead to higher decreases in THC concentrations compared to products which are widely used within the population for hair washing or hair care. This could lead to the strange situation where a person tests negative in hair samples while consuming regularly and testing positive in urine controls.

## 5. Conclusions

It could be shown that THC hair concentrations could be decreased to lower concentrations or to non-detectability by using everyday hair care products. Therefore, it is recommended to complement abstinence controls using hair samples by urine analysis. Furthermore, due to these potential invisible manipulations, quantitative results of THC concentrations in hair should be interpreted with caution regarding the conclusion that an accused individual is a seldom, regular or non-regular user.

## Figures and Tables

**Table 1:** Results of hair analysis before and after extraction.

Case Number	Product	Untreated Sample [ng/mg]	Treated Sample [ng/mg] *	Reduction [%]	Average Reduction [%]
1	vodka Gorbatschow 1	0.156	0.0576	63	
2	vodka Gorbatschow 2	0.0704	0.0351	50	
3	vodka Gorbatschow 3	0.798	0.332	58	
4	vodka Gorbatschow 4	0.216	0.128	41	
5	vodka Gorbatschow 5	0.0816	0.0302	63	
6	vodka Gorbatschow 6	0.0973	0.0211	78	
7	vodka Gorbatschow 7	0.0461	0.0228	51	
8	vodka Gorbatschow 8	0.680	0.219	68	
9	vodka Gorbatschow 9	0.0453	**<LoD**	NA	58
10	vodka Gorbatschow 10	0.395	0.133	66	
11	vodka Gorbatschow 11	0.0701	0.0317	55	
12	vodka Gorbatschow 12	0.585	0.196	66	
13	vodka Gorbatschow 13	0.106	0.0469	56	
14	vodka Gorbatschow 14	0.0441	**<LoD**	NA	
15	vodka Gorbatschow 15	0.0558	0.0299	46	
16	vodka Gorbatschow 16	0.0171	**<LoD**	NA	
17	vodka Gorbatschow 17	0.0762	**<LoD, approx. 0.0175**	77	
18	vodka Gorbatschow 18	0.178	0.110	38	
19	vodka Gorbatschow 19	0.457	0.195	57	
20	Seborin hair tonic 1	0.156	0.0592	62	
21	Seborin hair tonic 2	0.0704	0.0236	66	
22	Seborin hair tonic 3	3.27	0.399	88	
23	Seborin hair tonic 4	0.308	0.104	66	
24	Seborin hair tonic 5	0.0307	**<LoD, approx. 0.0177**	42	
25	Seborin hair tonic 6	0.0207	**<LoD**	NA	63
26	Seborin hair tonic 7	0.659	0.2220	66	
27	Seborin hair tonic 8	0.0451	**<LoD**	NA	
28	Seborin hair tonic 9	0.0362	0.0310	14	
29	Seborin hair tonic 10	0.178	0.0294	83	
30	Seborin hair tonic 11	0.457	0.0820	82	
31	Zydot 1	0.0987	0.0489	50	
32	Zydot 2	0.370	0.239	35	
33	Zydot 3	0.182	0.0512	72	52
34	Zydot 4	0.0230	<LoD,	NA	
35	Zydot 5	0.0394	**<LoD, approx. 0.0198**	50	
36	Zydot 6	0.0650	0.0293	55	
37	Desderman disinfectant 1	0.178	0.0598	66	
38	Desderman disinfectant 2	0.457	0.112	75	
39	Desderman disinfectant 3	0.156	0.0329	79	
40	Desderman disinfectant 4	0.0704	0.0333	53	
41	Desderman disinfectant 5	0.892	0.590	34	
42	Desderman disinfectant 6	0.0468	**<LoD**	NA	65
43	Desderman disinfectant 7	0.0929	**<LoD, approx. 0.0164**	82	
44	Desderman disinfectant 8	0.356	0.179	50	
45	Desderman disinfectant 9	0.0174	**<LoD**	NA	
46	Desderman disinfectant 10	0.150	0.0223	85	
47	Desderman disinfectant 11	<LoD	**<LoD**	NA	
48	Head & Shoulders 1	0.156	0.0985	37	
49	Head & Shoulders 2	0.0704	**<LoD, approx. 0.016**	77	
50	Head & Shoulders 3	0.080	0.0388	52	
51	Head & Shoulders 4	0.240	0.0869	64	52
52	Head & Shoulders 5	0.0800	0.0611	24	
53	Head & Shoulders 6	0.178	0.153	14	
54	Head & Shoulders 7	0.457	0.195	57	

* Concentrations < 0.02 ng/mg after extraction are shown in bold. In total, the THC concentrations in the 54 hair samples before and after treatment with commercially available everyday products and products advertised on the internet to be able to reduce the concentrations of drugs in hair are given. Please note that concentrations below the lower limit of detection (LoD) were set to 0 ng/mg in the statistical analysis. NA, not applicable.

## Data Availability

The original datasets are available on request from MVZ Dr. Stein + Kollegen for 5 years after publication.
